# Government stakeholders’ perspectives on the family planning environment in three Nigerian cities: qualitative findings from the Nigerian Urban Reproductive Health Initiative (NURHI) Sustainability Study

**DOI:** 10.1080/16549716.2020.1847821

**Published:** 2020-12-02

**Authors:** Courtney McGuire, Lisa M. Calhoun, Tolulope Mumuni, Amelia Maytan-Joneydi, Mojisola Odeku, Ilene S. Speizer

**Affiliations:** a Carolina Population Center, The University of North Carolina at Chapel Hill, Chapel Hill, NC, USA; b Center for Population and Reproductive Health, University of Ibadan, Ibadan, Nigeria; c Nigerian Urban Reproductive Health Initiative, Abuja, Nigeria; d Gillings School of Global Public Health, The University of North Carolina at Chapel Hill, Chapel Hill, NC, USA

**Keywords:** Reproductive health, institutionalization, donor funding, government officials, Nigeria

## Abstract

**Background**: The Nigerian government has made numerous commitments to expanding access to family planning services for its population yet has faced many challenges in implementing these commitments. Foreign donors provide support for expanding access to family planning in key populations.

**Objective**: This study examines the family planning environment after donor funding has ended, including how government stakeholders perceive family planning services and their role in providing them post donor funding.

**Methods**: The NURHI Sustainability Study used qualitative data to evaluate the sustainability of the Nigerian Urban Reproductive Health Initiative (NURHI), which focused on increasing the use of modern contraceptive methods, particularly among the urban poor. This study presents results from in-depth interviews with 16 key government stakeholders, selected using purposive sampling methods, in three cities: Ilorin (where NURHI Phase 1 programming discontinued in 2015), Kaduna (where programming continued under NURHI Phase 2), and Jos (a comparison city). A thematic analysis was employed to identify key themes related to government stakeholders’ perspectives on the family planning environment and sustainability of NURHI programming.

**Results**: Respondents from all three cities highlighted local political leaders’ positive perceptions about family planning. All respondents were open to continued foreign donor support for family planning services while respondents in Kaduna and Jos emphasized the need for governments to lead efforts among all family planning actors. Stakeholders highlighted the benefits of a dedicated and implemented family planning budget line and encouraged continued state financial support. Respondents in Kaduna and Ilorin praised the positive influence of NURHI programming while those from Ilorin reflected on the need for future programs to gradually close-out their efforts to support sustainability.

**Conclusions**: As donors look to transition to government ownership of family planning efforts, it is important for family planning programs to understand and incorporate government stakeholders’ perspectives into their sustainability planning efforts.

## Background

Nigeria’s population more than doubled in size from 1990 to 2019 and is expected to grow by 200 million from 2019 to 2050, an absolute growth in population numbers only exceeded by India [1]. The Nigerian government faces many challenges in ensuring that such a large population is healthy and educated, highlighting the need for quality family planning (FP) services. In 2018, 10.5% of all women of reproductive age in Nigeria were using a modern contraceptive method [2]. As part of their FP2020 commitment, the Nigerian government pledged in 2012 to achieve a modern contraceptive prevalence rate (mCPR) of 27% among all women of reproductive age by 2020 [3]. Projections indicate that Nigeria is not on track to meet this ambitious goal since the 2020 estimated prevalence is 14.8% [4]. As Nigeria recalibrates its post-FP2020 commitments and strategy, it is vital to recognize and understand perspectives of government leaders in creating and supporting an efficient and sustainable FP environment.

Sustainability, defined by Proctor as ‘the extent to which a newly implemented treatment is maintained or institutionalized within a service setting’s ongoing, stable operation’ [5] and by Scheier and Dearing as ‘the continued use of program components and activities for the continued achievement of desirable program and population outcomes’ [6], is an important yet poorly researched component of a program’s impact [7]. While most reviews of studies assessing the sustainability of health interventions have been focused in higher-income settings [6,8], a recent review focused exclusively on studies conducted in lower-income settings in sub-Saharan Africa [9]. Iwelunmor and colleagues reviewed 41 studies focused on sustainability of health interventions in sub-Saharan Africa, with only four focusing on reproductive health outcomes.

Programs that fail to sustain beyond the life of the program can have negative impacts on the health outcomes they were originally targeting by damaging trust with communities and organizations [10]. While many factors impact sustainability, community ownership can be a key facilitator to sustainability by supporting the trust that was built during the initial intervention. Multiple studies included in Iwelunmor and colleagues’ 2016 review noted the sense of pride that communities felt when they were involved in implementing and continuing an intervention. Ensuring that key stakeholders are involved throughout the program can also positively influence social norms to improve future uptake of the health intervention [9].

A key factor in many reviews of sustainability is the role of funding. Scheier and Dearing highlighted the two routes to sustainable funding: (a) institutionalization of efforts or (b) continued external financial support through alternate donors [6]. Iwelunmor and colleagues noted that a major barrier to sustainability was lack of financial leadership, including a lack of long-term financial planning and a reliance on external funds during and after programming activities [9]. Scheier’s review noted the pervasive perception among stakeholders that securing additional funding was critical for sustainability with some expressing that this was the preeminent factor that designated sustained programming [7]. Wiltsey and colleagues’ review of 125 studies on sustainability noted that qualitative studies frequently emphasized the engagement of key stakeholders and funding as strong influences on sustainability [8].

Over the past decade, the Nigerian government has established new mechanisms of financial support to reach FP goals with varying levels of success. In 2018, an estimated USD 546 million was needed to provide FP services for all Nigerian women who desired to prevent a pregnancy [11]. This represented a USD 478 million gap between the cost to provide services for women already using a method and those desiring a method [11]. In support of FP access for all, in 2011, the government of Nigeria removed the fees for FP commodities and committed to making its first contributions to the commodities budget, which had previously been solely donor driven [12]. In 2017, at the FP Summit in London, the Nigerian government updated its 2012 commitment to include USD 4 million annually for the procurement of contraceptives [13], yet only a quarter of that new allocated amount was spent in 2017 [14]. In 2018, the Nigerian government achieved this pledge by dispersing USD 4 million to UNFPA as counterpart funding to provide FP commodities. Yet, the 2019 Appropriations Bill that was signed by President Buhari in May 2019 dramatically slashed FP allocations [15]. It zeroed out counterpart funding from USD 4 million in 2018 and cut total available budgeted funds from USD 8 million in 2018 to USD 832,000 in 2019 [15]. Enhancing donor-funded FP programs requires the Nigerian government to consistently uphold their responsibility to provide counterpart funds [16] while ensuring a dedicated and implemented FP budget line at the federal and state levels [17].

Previous qualitative research has examined Nigerian government stakeholders’ perspectives on FP programming and policies [18–20] and government stakeholders’ perspectives on the long-term sustainability and financing of various health programs, including health systems strengthening and maternal and child health services [19,21,22]. Yet there has been little research on government stakeholders’ perspectives on the FP environment and how actors influence the sustainability of programming efforts. The goal of this study is to understand key government stakeholders’ perceptions of the FP environment in three Nigerian cities with a focus on examining the sustainability of the Nigerian Urban Reproductive Health Initiative (NURHI) program activities (see description below). Findings from this study can be used to inform the design and implementation of future FP programs that have the potential for long-term sustainability.

## Methods

### The Nigerian Urban Reproductive Health Initiative

In 2009, the Bill & Melinda Gates Foundation (BMGF) funded the Urban Reproductive Health Initiative (URHI) with the goal to expand modern contraceptive use with a particular focus on the urban poor in four countries: Nigeria, Kenya, Senegal, and India. From 2009 to early 2015, the Nigerian Urban Reproductive Health Initiative (NURHI) operated in six cities: Abuja, Ibadan, Ilorin, Kaduna, Benin, and Zaria. NURHI received additional funding to continue activities in Kaduna State (2015–2020), Oyo State (2015–2018), and launch activities in Lagos State (2015–2020). NURHI had five objectives: 1) integrate FP into other health services; 2) improve the quality of FP services; 3) build private-sector partnerships; 4) increase demand for FP services; and 5) advocate for an improved policy environment [23]. NURHI’s advocacy work included forming advocacy groups that developed coalitions to advocate to local governments for funding and provide mentoring support for government officials to better advocate for dedicated and released FP budget lines.

The Measurement, Learning & Evaluation (MLE) project, based at the authors’ institution, was initially funded to evaluate the URHI programs in each of the four countries and then received additional support from BMGF to evaluate the sustainability of NURHI Phase 1 programming in Nigeria through the NURHI Sustainability Study. The NURHI Sustainability Study’s objectives were to use qualitative and quantitative data to examine the lasting influence of NURHI activities in a city where NURHI had ceased operations after Phase 1 (Ilorin, Kwara State) as compared to a city where it continued in Phase 2 (Kaduna, Kaduna State) and a city where NURHI had never operated (Jos, Plateau State). Household and facility-based surveys were undertaken in late 2017 followed by qualitative interviews in July 2018. This study uses data from in-depth interviews with influential government leaders to understand the FP environment in each of the study cities in 2018, three years after the program ended in Ilorin.

### Recruitment and data collection

As part of the NURHI Sustainability Study, in-depth interviews were conducted with 16 key government stakeholders. Sixteen interviews across the three sites were deemed sufficient to achieve the study’s objective; however, additional informants were identified, and the study team was ready to recruit them if it was deemed that important study themes were not addressed with sufficient detail and new areas were still being uncovered. As interviews were conducted, study team members discussed the findings and reviewed them for signs of saturation. Five interviews were conducted in Ilorin, five in Jos and six in Kaduna. The stakeholders were recruited using purposive sampling methods. Stakeholders in Ilorin and Kaduna were identified by NURHI’s advocacy core group as individuals that they had worked closely with and who had a keen understanding of NURHI’s activities. Additionally, NURHI helped identify key government stakeholders involved in reproductive health activities in Jos. Study team members contacted the selected stakeholders via initial phone calls to ask them to participate in the study. They scheduled appointments for the interview, which was then conducted in the stakeholders’ office to ensure privacy and confidentiality. Key informants held positions such as state or local government area reproductive health coordinator, commissioner for health, and FP manager.

The in-depth interview guide was jointly developed by the study teams at the authors’ institutions. The guides were translated into Hausa and Yoruba, the predominant local languages in Kaduna/Jos and Ilorin respectively, and then back translated to ensure the original meanings were retained. The interview guide was pre-tested with government stakeholders in Ibadan, a NURHI Phase 1 city not included in the NURHI Sustainability Study; the guide was revised based on feedback from interviewers and respondents. The interview guide included seven questions with up to five probing follow-up questions that covered topics related to sustainability of FP programming including government support of FP, local FP champions, foreign donor support, and future expectations of FP within the city. Respondents were asked to reflect on changes they had seen on these topics within their cities over the last five years. They were also asked to comment on specific recent events that had happened in their cities, such as recent increases in FP budget lines or recent challenges with implementing FP budget lines. Contact the first author for a copy of the interview guide.

Interviewers experienced in FP qualitative research were recruited and trained by the Center for Population and Reproductive Health (CPRH), located at an author’s institution, to collect the data. Following a verbal informed consent process, interviews were conducted in the language that the informant felt most comfortable with. Verbal consent was used to avoid having written names of participants and to increase participants comfort level with responding to the questions. Interviews lasted between 22 and 92 minutes and were audio-recorded.

### Data analysis

The CPRH study team led the transcription and translation process from these recordings using ATLAS.ti (v.7). For this analysis, two MLE team members (CM and AMJ) performed a thematic analysis of the in-depth interviews from April to June 2019. This involved multiple readings of the transcripts to identify emerging themes and then developing a codebook for analysis. The two coders independently and iteratively coded each transcript. Throughout the coding process, they continued to update and refine the codebook. Following coding, they reviewed the resulting themes and each developed a Microsoft Excel matrix that was used to capture the themes across the rounds of coding. The coders then reviewed each matrix to discuss any discrepancies and agreed upon a final version. Identifying information was not included in the final presentation of results to protect respondent’s confidentiality.

### Ethical approval

Ethical approval was obtained from the National Health Research Ethics Committee of Nigeria (NHREC/01/01/2007) and the University of North Carolina at Chapel Hill’s Institutional Review Board (#17-1215). Additional approvals were obtained from the Commissioners of Health within each state.

## Results

Stakeholders in all three cities had a positive perception of government’s role in FP. They highlighted their government’s increasing awareness of the importance of FP efforts. Respondents felt that dedicated budget lines were examples of high levels of support while all respondents, especially those in Ilorin, emphasized the need to implement the planned budget lines. Government stakeholders were also adamant about the continued support of foreign donors. Kaduna and Jos stakeholders emphasized the expectation that foreign donors will play a supportive rather than leadership role in providing FP services while stakeholders in Ilorin were more focused on re-establishing connections with foreign donors. Stakeholders in Kaduna and Ilorin praised NURHI’s efforts in their cities and Ilorin stakeholders reflected that a more gradual program close out would have better positioned the government to institutionalize NURHI programs. Below we explore these themes in more depth.

### Changes in government’s perceptions of family planning

Overall, respondents felt that their local and state governments supported FP provision within their city. When asked about the change in perception over the last five years and since the end of NURHI Phase 1, respondents from Kaduna were the most effusive in their praise for how support has grown and strengthened. One respondent noted:

“*I will [say it has grown] amazingly, surprisingly. There has been significant change in perception of family planning among the leaders.”* (Kaduna stakeholder)

Respondents in Kaduna felt that government stakeholders had recognized the importance of FP interventions for minimizing maternal mortality and morbidity and had thus committed themselves to supporting FP efforts. One respondent shared:

“*We have come to accept family planning as one of the solutions to the high maternal death we are experiencing, especially in this part of our country*.” (Kaduna stakeholder)

In Ilorin, a city where NURHI ceased operations in March 2015, respondents were more mixed in their perception of government support for FP and how it had changed over the past five years. Respondents in Ilorin shared that there had been an increase in support for FP efforts in the past five years but noted that local budget constraints had led to a decrease in financial commitment for FP.

### Financial support for family planning

#### Dedicated budget lines

Throughout the interviews, respondents from all three cities emphasized the importance of a dedicated FP budget line within the state’s budget. Respondents felt that it was the government’s duty to provide adequate funding for FP and were adamant that this money be protected within the budget. In Jos, respondents were positive about recent changes to implement separate budget lines for FP. One Jos respondent shared:

“*We have a separate budget line and we, the government, were able also to buy consumables, which has been our problem in the past*.” (Jos stakeholder)

In Kaduna, respondents expressed the significance they placed on the change in the government’s efforts to secure and implement budget lines, as demonstrated by one respondent:

*“It was just only in the last few years that the state had a budget line for family planning. In the state, there is no greater commitment for government than to have a budget line purposefully for family planning.”* (Kaduna stakeholder)

Respondents in Ilorin expressed more frustrations about the process of securing and implementing a FP budget line and called upon the government to ensure adequate funding. One respondent commented:

“*It is not a matter of announcing budgets and the thing is not implemented. But if you budget for something, implement it so that it will affect the people of the state.”* (Ilorin stakeholder)

#### Foreign donors and family planning

Respondents in all three cities expressed positive outlooks on foreign donor’s presence within their city’s FP environments and encouraged continued engagement. One respondent in Jos noted:

“*Donors have been the key actors in supporting family planning services. I will say without the donor we would not have achieved what we have achieved because the trainings are mostly done by donors. The availability of commodities, providing commodities is mostly done by donors.”* (Jos stakeholder)

Within Ilorin, respondents were positive about foreign donors in general but felt that their involvement in the state had waned in recent years. In response to the role of foreign donors with their city, multiple respondents in Ilorin expressed the desire for foreign donors to support local FP efforts, including capacity building, outreach, and monitoring. One stakeholder shared:

*“They should assist us by training and the re-training and the second one is supplying us with this consumable, so that we will be free as a whole.”* (Ilorin stakeholder)

Respondents in Kaduna and Jos seemed more confident of their foreign donors’ support and shared that they felt strongly that foreign donors should work with the government to provide FP services. They felt that the government should lead these activities and donors should assume a more supportive role. Respondents shared:

“*The role of the foreign donors is to support the government to implement the services, financial support*.” (Kaduna stakeholder)

*“For the foreign donor, of course they go a long way to help. We need help from others to support us. So, they come through the government and we work together with them.”* (Jos stakeholder)

#### Perceptions of NURHI, family planning advocacy and family planning sustainability

In Ilorin and Kaduna, respondents praised NURHI’s program activities such as demand creation; advocacy to government, traditional, and religious leaders; and service provision. Ilorin respondents reflected on how the end of NURHI activities affected the local FP environment, including advocacy to local governments and community members. One respondent noted:

“*[Advocacy to the community] is not the same thing because [when] NURHI was around we normally did advocacy to many places, including in the schools, but since they left, there is no such thing again*.” (Ilorin stakeholder)

Stakeholders from all three cities identified and praised local advocacy groups that were working to raise support for dedicated FP budget lines. Support for advocacy initiatives from external organizations was noted within all three cities. In Ilorin, stakeholders highlighted NURHI’s Advocacy Core Group and its transition to being supported by other external organizations. One stakeholder shared:

“*Even it is- that group [NURHI’s Advocacy Core Group] is still operating now. So, we have the group that does advocacy. So, we go from local government to local government, at least to create awareness and any local government that has a problem, at least they try to come in and render help.”* (Ilorin stakeholder)

In Kaduna, stakeholders noted the positive changes in attitudes towards FP among key stakeholders and the continued use of NURHI’s advocacy kits. One respondent noted:

*“In the last five years, the intensive advocacy by those who are supporting us driving family planning particularly NURHI. [They] activate a lot of change in perception of family planning by intensive advocacy.”* (Kaduna stakeholder)

Respondents from Jos noted various groups conducted advocacy to government stakeholders and community leaders. One respondent shared that their advocacy groups were targeting their outreach efforts to key elites, including the wives of government leaders, so that these individuals could then encourage stronger FP policies. Others discussed advocacy targeted directly at government officials.

*“On the Plateau, we have an advocacy core group which is called Voice for Family Planning Reproductive Health Center whose focus is advocacy to government leaders to invest in family planning. This body has activities and government leaders are beginning to understand the need to key in and ensure that those who desire to use contraception or contraceptive methods have it.”* (Jos stakeholder)

#### Perceptions of sustainability

Stakeholders in Ilorin shared some frustrations with how the end of programming rolled out for their city and expectations for how future programs will close out. They emphasized the need for government and foreign donors to continue to partner until the government can adequately institutionalize the programming efforts. Two stakeholders noted:

“*When you give us a program, after with the period has elapsed, you will not just leave the program and just go like that. You go gradually, gradually, and gradually. What will sustain the program is very necessary.”* (Ilorin stakeholder)

*“The state government should be able to sustain the program before that time. But at the same time, we still want the foreign donors to assist us but in a broader way.”* (Ilorin stakeholder)

## Discussion

This study capitalized on a natural experiment linked to the end of programming in one city (Ilorin) and the continuation within a second city (Kaduna). Through qualitative interviews with key government stakeholders in these two cities, along with a third comparison city (Jos), this study was able to reflect on the sustainability of FP programming efforts after donor funding ends and provide useful suggestions for future programs to incorporate into their family planning efforts.

Government stakeholders in all three cities were positive about their governments’ perspectives on FP and noted many examples of key political and religious leaders publicly commenting on the benefits of FP. Respondents praised the positive impact of advocacy groups on political leaders’ willingness to speak publicly. This aligns with Iwelunmor and colleagues’ findings from a systematic review of sustainability of health programs in sub-Saharan Africa that showed that when key stakeholders are involved in implementing interventions and encouraging community support, these programs are more likely to sustain due to changed community social norms and increased usage [9].

Respondents emphasized the need for state and federal governments to provide financial leadership and support for FP efforts. While many studies highlight the importance of institutionalization of programming efforts [5,6,9], continued external funding may also be indicative of a lower level of sustainability. Within all three cities, respondents’ openness and eagerness to partner with foreign donors and NGOs highlights the potential for slowly reducing funding to allow additional time for governments to institutionalize external programming efforts. Of note, stakeholders from Jos and Kaduna emphasized foreign donors allowing governments to lead the partnership, suggesting an openness to increased institutionalization of efforts whereas stakeholders in Ilorin were focused on reestablishing and strengthening partnerships with foreign donors.

One important component of sustainability of health programs is the maintenance of community-level partnerships or coalitions developed during the donor funded program [6]. Respondents from all three cities mentioned the role of advocacy groups in influencing key stakeholders in the FP environment; this aligns with other research on the importance of advocates on FP policies and budgets [24,25]. Respondents in Ilorin were pleased to mention the continued efforts of advocacy groups, now supported through other non-governmental organizations. These advocacy groups represent a strategy toward maintaining activities launched under NURHI Phase 1 programming through external support which has been shown to be important for the sustainability of health programming [6], while also representing the community’s support of program efforts.

Iwelunmor and colleagues (2016) noted the lack of financial leadership as a key barrier to sustainability. Respondents in all three cities noted the government’s responsibility to institutionalize programming efforts by providing financial support through dedicated and released FP budget lines. While the Kwara State (Ilorin) government announced a dedicated FP budget line item [26], it had not released the funding; this represents a challenge with moving towards institutionalization of program efforts in Ilorin. Ilorin’s experience is in comparison with Kaduna’s where NURHI Phase 2 continued to be a strong partner in advocating for FP budget lines. Kaduna State included FP budget lines in previous budgets and earmarked 165 million Naira for the 2019 budget [27]. Continued advocacy has resulted in growing budgetary support for FP in Kaduna, hopefully leading to institutionalization of programming to minimize the impact of NURHI Phase 2 close out in 2020.

Jos, the NURHI Sustainability Study’s comparison city, has benefitted from the efforts of externally funded advocacy groups, such as Advance Family Planning (also BMGF funded). These groups advocated in Plateau State for a dedicated FP budget resulting in the state’s first dedicated FP budget line of 5 million Naira in 2016 [28], which was released the following year [29]. In around 2018, Plateau State joined The Challenge Initiative, a BMGF-supported program designed to build upon the successes of NURHI and encourage local ownership at the outset. Local advocacy groups’ continued efforts to encourage dedicated and implemented FP budget lines will contribute to increased institutionalization and minimize the impact of cessation of NURHI and other donor funded FP programs.

While there has not been extensive research on key stakeholders’ perspectives of the sustainability of FP efforts in Nigeria, there has been research into stakeholders’ perspectives on the sustainability of HIV/AIDS services in Nigeria [30–32]. Itiola and Agu’s 2018 qualitative study on country ownership and sustainability of Nigeria’s HIV/AIDS supply chain system was conducted with key stakeholders representing each geopolitical zone of the country to better understand what happens when large donors decrease their levels of funding for key health issues. Findings from our study align with many of those shared in Itiola and Agu’s qualitative work with both studies highlighting the important role governments play in providing overarching leadership and the ongoing need for advocacy efforts that encourage this leadership to grow. The biggest challenge noted by Itiola and Agu’s key stakeholders was inadequate domestic funding for HIV/AIDS supply chain systems while respondents in Ilorin shared a similar perception during this study, noting the lack of release for FP budget line funds. As the literature on government stakeholders’ perspectives on FP sustainability in Nigeria is sparse, programs and policy makers can learn from the HIV/AIDS sector on transitioning to country ownership and examining the long-term sustainability of originally donor-funded projects.

## Limitations

This study is not without limitations. The study utilized purposive sampling to select key government stakeholders to interview. Respondents were selected from a list of proposed government stakeholders provided by NURHI staff members. While this ensured the study team spoke with stakeholders who had a keen understanding of NURHI activities in their city, it also increased the chances of response bias within our data in favor of the program. Furthermore, respondents, especially those from Ilorin, could have responded to the discussion with a specific aim to encourage future funding. While interview guides were developed to ask questions regarding various components of sustainability, respondents seemed to heavily focus on the financial aspect of sustainability. Moreover, interviews with more stakeholders, including representatives of organizations that took over NURHI programming efforts in Ilorin such as Pathfinder International, would have provided richer context for the sustainability of these programs. Additionally, the overall NURHI Sustainability Study was not designed to allow for triangulation with other data sources.

Political support and funding for FP programs are constantly evolving and highly variable. This study represents a specific time period for the three study cities and is not able to track these evolving perspectives. Finally, as with most qualitative studies, we did not intend this study to be representative of the population or all government stakeholders. Therefore, we cannot generalize the findings beyond the study cities or specific stakeholders interviewed. However, we believe that the voices highlighted in this study reflect many of the ongoing challenges and successes of FP programming for the long-term in Nigeria.

## Conclusions

Long-term program sustainability remains a critical focus of the global FP community [33]. This study emphasizes the leading role that key government stakeholders in three Nigerian cities believe their governments should play in their city’s FP environment. While institutionalization of NURHI Phase 1 activities in Ilorin has been limited, there are many components of programming that have been continued through other external funders and implementing partners. While this does not represent sustainability, it does offer the city-level government an extended opportunity to institutionalize donor funded FP activities. Government stakeholders in Ilorin should continue to institutionalize NURHI programming efforts by fully funding and implementing FP budget lines. While those in Kaduna should heed Ilorin’s experience and continue to nurture advocacy efforts to support, implement, and increase budget lines to institutionalize NURHI Phase 1 and 2 efforts more efficiently. A gradual shift away from donor funds and a concurrent increase in the government’s leadership of project activities will allow for successful institutionalization and sustainability. Lessons learned from cessation of NURHI Phase 1 activities in Ilorin should also be heeded by stakeholders in Kaduna where NURHI Phase 2 activities ended in 2020.

## Paper context

Few studies have been conducted to evaluate the sustainability of a program following the cessation of donor funding. This study contributes to our understanding of the sustainability of FP programs by presenting key government stakeholders’ perspectives on the FP environment and donor funded programs in Nigeria. The findings are a call to action for all donor funded programs to further engage government stakeholders during all steps of programming, which will encourage long-term sustainability.
